# Prevalence, correlates, and health associations of 24-hour movement behaviours in Slovenian adults: a protocol for a longitudinal population-based study

**DOI:** 10.1186/s44167-025-00073-z

**Published:** 2025-03-19

**Authors:** Kaja Kastelic, Tjaša Knific, Nejc Šarabon

**Affiliations:** 1 Andrej Marušič Institute, University of Primorska, Muzejski trg 2, Koper, 6000 Slovenia; 2 InnoRenew CoE, Livade 6a, Izola, 6310 Slovenia; 3 National Institute of Public Health, Trubarjeva cesta 2, Ljubljana, 1000 Slovenia; 4 Faculty of Health Sciences, University of Primorska, Polje 42, Izola, 6310 Slovenia

**Keywords:** Time-use epidemiology, Longitudinal study, Population-based study, Population surveillance, Compositional data analysis, Physical activity, Sedentary behaviour, Sleep, Well-being

## Abstract

**Background:**

Physical activity, sedentary behaviour, and sleep (i.e., 24-hour movement behaviours) are among key determinants of health and well-being. However, epidemiological studies that investigate these behaviours while accounting for their co-dependent nature are still scarce. This article describes the protocol of a population-based study that aims to explore the prevalence and correlates of 24-hour movement behaviours among Slovenian adults and their cross-sectional and longitudinal associations with health and well-being.

**Methods:**

Participants will be recruited through the National Health-related Lifestyle Survey study that is conducted among a national representative sample of Slovenian adults aged 18–74 years (*n* of invited participants every four years = 17,500). Baseline data collection will include assessment of 24-hour movement behaviours using activPAL accelerometers. Domain-specific movement behaviours, built environment, and well-being will be assessed using questionnaires. A linkage to the National Health-related Lifestyle Survey that include data on demographics (e.g., marital status, socio-economic status), health-related behaviours (e.g., diet, smoking), and health (e.g., self-reported health, comorbidities) will also be established. The follow-up data collections (every four years) will include self-reported assessments of 24-hour movement behaviours, built environment, health and well-being, and linkage to the national health-related registers.

**Discussion:**

The study will produce new knowledge on 24-hour movement behaviours, their socio-demographic and built environment correlates, and their cross-sectional and longitudinal relationship with health outcomes using compositional data analysis. It will reveal an insight into the relative importance of domain-specific and type-specific movement behaviours, informing future 24-hour movement guidelines for adults.

## Background

Physical activity, sedentary behaviour, and sleep (i.e., 24-hour movement behaviours) are among key determinants of health and well-being. Studies have shown that more physical activity, less sedentary behaviour, and enough sleep are associated with a lower risk of chronic non-communicable diseases, including cardiovascular diseases, type 2 diabetes, obesity, some cancers, anxiety and depression as well as premature mortality [1–4].

Previous studies mostly investigated health effects of a single movement behaviour in isolation [1–4]. However, given that data on time spent in physical activity, sedentary behaviour, and sleep are co-dependent components of a whole (e.g., 24-hour day), researchers have become aware that these behaviours should be examined in relation to each other and that nature of these data require specific analytical approaches [5, 6]. This awareness also led to the paradigm shift towards conceptualising physical activity, sedentary behaviour, and sleep as inseparable components of a whole that should be addressed in a combination [5, 7–10].

The novel paradigm was soon recognized in the field [11–14]. There has been important progress in the development of analytical approaches for compositional data [15, 16] that supports empirical studies on 24-hour movement behaviours and health [11–14]. However, recent reviews revealed that especially longitudinal studies exploring relationships of 24-hour movement behaviours with a wide range of socio-ecological correlates and health outcomes are scarce [11–14].

In recent years, the importance of the 24-hour movement paradigm has also been recognised by government authorities who develop public health guidelines. Several countries [8, 9, 17–21] as well as the World Health Organisation [22] have released 24-hour movement guidelines that integrates recommendations on physical activity, sedentary behaviour, and sleep. Due to the lack of evidence on the combined health effects of 24-hour movement behaviours, the first generation of the 24-hour movement guidelines was largely informed by the studies that explored health effects of a single movement behaviour in isolation. As such, the authors of the Canadian 24-h movement guidelines for adults [9] indicated that more research with stronger designs (e.g., longitudinal design, device-measured movement behaviours), as well as examining different dimensions of movement behaviours is needed. For example, there is a lack of evidence on the relative importance of domain- and type-specific physical activity and sedentary behaviours, and on the importance of sleep consistency, napping, and daytime alertness [9].

In 2023, our research team started a project that aims to implement the concept of 24-hour movement behaviours as a determinant of health into the Slovenian environment, creating an opportunity to establish a new population-based study on device-measured 24-hour movement behaviours among Slovenian adults (*the GIB study*). Given that the Slovenian 24-hour movement guidelines for adults are under development, it is important to establish and monitor prevalence of (un)healthy 24-hour movement behaviours among Slovenian adults and to identify its correlates and determinants that will help inform future evidence-based targeted interventions.

To summarise, the objective of *the GIB study* is to (*i*) estimate the levels of device-measured and self-reported 24-hour movement behaviours among Slovenian adults, (*ii*) to identify socio-demographic and built environment correlates and determinants of (un)healthy levels of 24-hour movement behaviours, and (*iii*) to explore cross-sectional and longitudinal associations of 24-hour movement behaviours (including domain-specific and type-specific movement behaviours) with health outcomes while accounting for the co-dependency of data on 24-hour movement behaviours.

## Methods

### Participant recruitment and study design

This will be a longitudinal population-based epidemiological study on 24-hour movement behaviours, built environment, and health. Participants will be recruited through the National Health-related Lifestyle Survey study that is conducted by the National Institute of Public Health (NIJZ). Every four years, the Republic of Slovenia Statistical Office prepare national representative sample (based on the Central Population Register, the sampling frame present Slovenian citizens and foreigners who have residence in Slovenia, using stratified simple random sampling design and the strata being region and settlement type of the residence) of 17,500 Slovenian residents aged 18 to 74 years that is invited to participate in a national survey. The invitations are sent via post to their home addresses. The survey is usually open between May and September and the response rate is usually around 60%. This sample will be invited to also take part in the prospective study on device-measured 24-hour movement behaviours (we expect that around 5% of the survey responders will also accept an invitation for the device-based study).

**Fig. 1:**
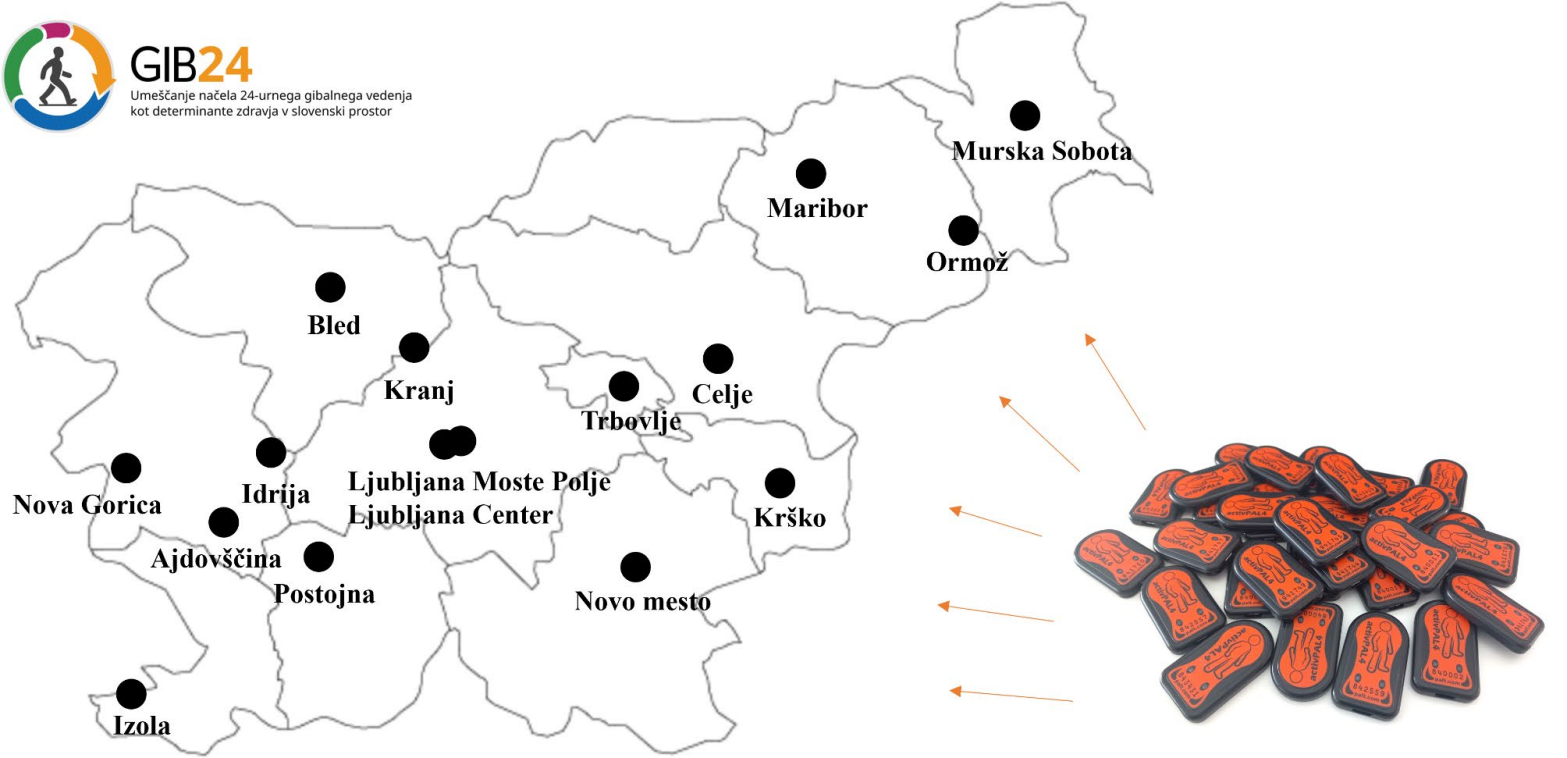
Locations of data collection of the GIB study in 2024

Interested participant will enter online appointment system for *the GIB study* by their unique code (sent by post) or will register by telephone. The appointment system will include all relevant information about *the GIB study* (including e-mail and telephone number for more information). Participants will choose a desired location, date, and time for their enrolment in *the GIB study*. There will be several options available (Fig. 1).

Participant will meet a member of the research team in groups of 5–10 participants. The researcher will introduce the study, and participants will have the opportunity to ask any question before signing an informed consent. Participant will be asked to complete two questionnaires on movement behaviours. The researcher will place an activPAL accelerometer on the participant’s thigh and ask the participant to wear it for full 7 days (24-hour per day), and to keep a sleep and work diary. Participants will receive additional adhesive dressing to attach the activPAL to their thigh and written instructions on how to change adhesive dressing if needed. Participants will be able to e-mail or phone the research team during the activPAL data collection period.

After 7 days, the participants will meet a member of the research team again. They will return activPAL and diary, and complete a questionnaire on movement behaviours (GIB24-Q), as well as questionnaires on built environment and well-being. Participants will receive an individual report of their 24-hour movement behaviours assessed by the activPAL, accompanied by 24-hour movement guidelines (delivered via e-mail or post).

**Fig. 2:**
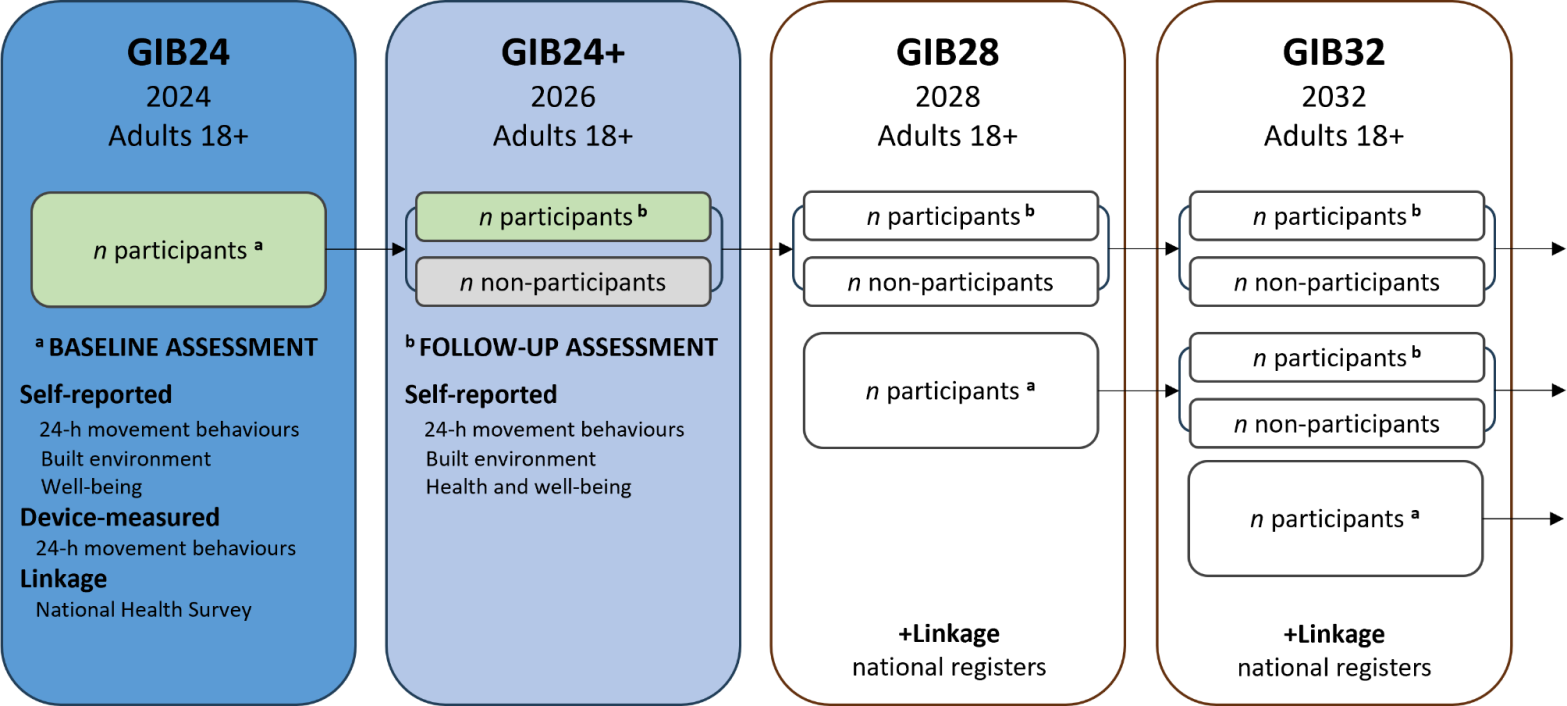
The SPIRIT figure: study design for the prospective GIB study

Every four years after the baseline assessment (except for the sample recruited in 2024 who will also be reinvited after 2 years), participants will be reinvited to complete questionnaires on movement behaviours, correlates, and health by e-mail or by post letter (see Fig. 2). To increase the adherence in the follow-up data collections, every 10th participant who completes the questionnaire will receive a voucher for 20 euros. For those not responding, two reminders will be sent. In 2028 we will establish a linkage to the national health-related register. All participants will receive an additional information about this and will be asked to provide informed consent to allow to linking their data with the national health-related register. Information regarding data safety will be also provided to the participants. The first recruitment of participants and baseline data collection was conducted in 2024, and those participants will be followed from the 2024 onwards (see Fig. 2 and Additional file 1 for SPIRIT checklist [23]).

The following recruitments of new participants are planned for every four years (in 2028, in 2032, etc.). Participants will undergo baseline assessment in the first year when entering the study and then be followed every four years (e.g., the second recruitment of participants and baseline data collection will be conducted in 2028, and those participants will be followed from the 2028 onwards). The GIB study has an ambitious to become a longitudinal study that will span more than a decade.

### Estimated sample size

The response rate for the National Health-related Lifestyle Survey is usually around 60% (*n* ~ 10,500). Of those that participate in the National Health-related Lifestyle Survey, we anticipate that around 5% of responders will also accept an invitation for *the GIB study* (*n* ~ 525 participants every four years). Based on the previous studies, we can expect that around 90% will provide at least 4 valid days of activPAL data, and that 1% of complete activPAL data loss may happen due devices lost by participants [24]. Based on our previous experiences, up to 5% of activPAL data loss may also happen due to activPAL technical failure during data collection. The dropout rate in the follow-up data collections may be as high as 30% [25]. Therefore, the expected number of participants from each baseline assessment who will provide at least 4 days of activPAL data is 472 participants (i.e., 472 participants recruited in 2024, 472 participants recruited in 2028, etc.), and for the follow-up 330 participants. An a priori power analysis determined that expected sample size is sufficient to detect a minimum effect size of 0.2 (80% power, α = 0.05) in our investigation of the relationships between 24-hour movement behaviours and the selected variables using multivariate regression [26, 27].

### Inclusion criteria

The inclusion criterion is participation in the National Health-related Lifestyle Survey (repeated cross-sectional survey conducted every four years) that includes a national representative sample of Slovenian adults aged 18 to 74 years.

### Measures

#### 24-hour movement behaviours

*Activity monitor activPAL4micro* (PAL Technologies, Ltd.) is a small and light tri-axial accelerometer (4 × 2 × 0.4 cm; 9 g) that is worn on the anterior aspect of the thigh. Participants will wear activPAL for seven consecutive days (24 h per day) and complete a sleep and work time diary [28]. The diary will collect information about the time when participants woke up and fell asleep, the time of daytime naps, and the time being awake during reported sleep period (if any). For employed participants, the diary will also collect data on time when getting to work and leaving work. Proprietary software PALconnect (PAL Technologies, Ltd.) will be used to initialise the activPAL (by activPAL4 default recording mode), and PALanalysis (PAL Technologies, Ltd.) will be used to download and process the data (by CREA algorithm using 24-hour protocol that allow up to four hours of non-wear time).

The sleep onset and offset will be manually adjusted within the PALanalysis by combining the information on time in bed from the CREA algorithm and diary reported sleep time, as suggested previously [29]. Sleep onset will be determined using diary entries unless the activPAL recorded a period of upright activity within 20 min after diary-reported sleep onset. In that case, sleep onset will be determined as the start of the first recorded non-upright event after that upright activity. Similarly, sleep offset will be determined based on diary entries unless the activPAL recorded a period of upright activity within one hour before diary-reported sleep offset. In that case, sleep offset will be determined as the end of the non-upright event before that upright activity (similar approach was used in previous studies [30, 31]).

The activPAL data will be exported using PALbatch (PAL Technologies, Ltd.) as “Daily summary outcomes” in a.csv format. Daytime napping duration from the sleep diary will be added to sleep time (and deducted from lying time while awake). Non-wear time will be proportionally reallocated to wake-time behaviours only, as suggested previously [32]. Data on posture-based time-use composition, including stepping, standing, sitting, lying while awake, and sleeping will be extracted. In addition, the activPAL data will be exported as “Events” in a.csv format and further processed in Rstudio using the package “*activpalProcessing*” [33] to obtain estimates on movement-based time-use composition, including moderate- to vigorous-intensity physical activity, light-intensity physical activity, sedentary behaviour, and sleep. For employed participants, we will also obtain estimates on the duration of occupational and non-occupational wake time posture-based and movement-based behaviours (based on diary reported work time, processed in R using a custom-made analytical code). Previous studies confirmed the validity of posture-based [34] and movement-based [35] activPAL estimates.

To assess self-reported movement behaviours, participants will complete a novel 10-item *GIB24-Q* questionnaire that asks about domain specific moderate- to vigorous-intensity physical activity (recreational, transportational, occupational, domestic), muscle strengthening exercise, balance exercise, walking time, sedentary behaviour, recreational screen time, and sleep in the usual week (Kastelic et al., in publication process). This questionnaire has been developed for the purpose of population surveillance of 24-hour movement behaviours in Slovenia. In 2024, it has been integrated in the National Health-related Lifestyle Survey (the validation study is in process).

To assess self-reported movement behaviours, participants will also complete a 16-item *Global Physical Activity Questionnaire* [GPAQ] that asks about domain specific moderate- to vigorous-intensity physical activity (work, transportational, leisure-time) and sedentary behaviour in the typical week [36]. The GPAQ is one of the most frequently used questionnaires for the assessment of physical activity and sedentary behaviour and it was shown to be valid and reliable [36].

#### Built environment

*Healthy Built Environment Questionnaire* [HeBEQ] will be used to assess the built environment, and the time spent in built and natural environments (Kastelic et al., in publication process). The HeBEQ is a novel 46-item questionnaire that assesses perceptions of neighbourhood (14 items), home (14 items), and work (13 items) environments. Respondents indicate their level of agreement or disagreement with each statement of built environment characteristics. The HeBEQ also asks about the time spent in these environments (3 items) as well as the time spent outdoor and specifically in the natural environment (2 items) in the past seven days. Responses to the questions on time-use in different environments are provided in hours and minutes per day (the validation study is in process).

Based on participant’s home address we will obtain information on neighbourhood walkability using *Walk Score*^*®*^*index* (https://www.walkscore.com), and several environmental indicators including air quality and number of days with high temperatures (http://rte.arso.gov.si/). The Walk Score index is an open access measure of neighbourhood walkability on a scale from 0 to 100, where values closer to 0 indicate lower walkability and values closer to 100 higher walkability. The index is calculated based on walking routes to destinations (e.g., grocery stores, schools, parks, restaurants, retail), road metrics (e.g., block length, intersection density), and population density [37]. Data sources include Google maps information. Walk Score was shown to be a valid measure of estimating neighbourhood walkability [38].

#### Well-being

Eight short questionnaires will be used to assess different aspects of physical and mental well-being:

- *WHO-5 Well-being Index* [39] is a 5-item questionnaire that assesses subjective mental well-being in the past two weeks. It includes statements that are rated on a 6-point scale (ranging from “at no time” to “all of the time”), and each statement is assigned 0–5 points accordingly. The total score ranges on a scale from 0 to 100 and is calculated by summing the scores on each of the questions and multiply by 4 (values closer to 0 indicate poor well-being and values closer to 100 best well-being). The WHO-5 was shown to have adequate validity for screening purposes as well as an outcome measure to assess well-being over time [39].
- *Need for Recovery Scale* [NRS] [40] is a 9-item questionnaire (with an unanchored recall period) that assesses need for recovery for employed individuals. It includes statements that are rated on a 5-point scale (ranging from “never” to “always”), and each statement is assigned 1–5 points accordingly. The sum of scores is converted to an index from 1 to 100, where values closer to 1 indicate lower requirement for recovery and values closer to 100 higher requirements for recovery. The NRS has a good test-retest reliability, sensitivity to detect change, and was shown to be a good predictor of long-term work-related fatigue symptoms and sickness absence [40].
- *International Fitness Scale* [IFIS] [41] is a 5-item questionnaire that assesses perceived fitness level when compared with peers (cardiorespiratory fitness, muscular fitness, speed–agility, flexibility, and overall fitness). It includes statements that are rated on a 5-point scale (ranging from “very poor” to “very good”). The IFIS showed adequate test-retest reliability and validity for use in epidemiological studies [41, 42].
- *Epworth Sleepiness Scale* [ESS] is an 8-item questionnaire that assesses daytime sleepiness [43]. The ESS is asking about the likelihood of a responder dozing off or falling asleep in common situations. It includes statements that are rated on a 4-point scale (ranging from “would never doze” to “high chance of dozing”), and each statement is assigned 0–3 points accordingly. The total score ranges on a scale from 0 to 24 and is calculated by summing the scores on each of the questions (values closer to 0 indicate lower daytime sleepiness and values closer to 24 excessive daytime sleepiness). The ESS showed high level of internal consistency [44] and adequate validity [45].
- *Fatigue Assessment Scale* [FAS] is a 10-item questionnaire that assesses usual symptoms of chronic fatigue, including physical and mental symptoms [46]. It includes statements that are rated on a 5-point scale (ranging from “never” to “always”), and each statement is assigned 1–5 points accordingly. The total score ranges on a scale from 10 to 50 and is calculated by summing the scores on each of the questions (values closer to 10 indicate lower fatigue and values closer to 50 severe fatigue). The FAS showed good internal consistency and validity [46].
- *Satisfaction With Life Scale* [SWLS] is a 5-item questionnaire that assesses global cognitive judgements of satisfaction with one’s life [47]. It includes statements that are rated on a 7-point scale (ranging from “strongly disagree” to “strongly agree”), and each statement is assigned 1–7 points accordingly. The total score ranges on a scale from 5 to 35 and is calculated by summing the scores on each of the questions (values closer to 5 indicate lower life satisfaction and values closer to 35 higher life satisfaction). The SWLS showed good internal consistency, test-retest reliability, and validity [47].
- *Perceived Time Poverty Scale* [PTPS] is a 6-item questionnaire that assesses individuals’ perceptions of lacking freely disposable time [48]. It includes statements that are rated on a 7-point scale (ranging from “strongly disagree” to “strongly agree”), and each statement is assigned 1–7 points accordingly. The total score ranges on a scale from 6 to 42 and is calculated by summing the scores on each of the questions (values closer to 6 indicate lower time poverty and values closer to 42 higher time poverty). The SWLS showed good internal consistency and validity [48].
- *Nordic Musculoskeletal Questionnaire* [NMQ] is a questionnaire that assesses musculoskeletal symptoms in nine body regions over the past 12 months (neck, shoulders, upper back, elbows, wrists/hands, lower back, hips/thighs, knees, and ankles/feet). It includes questions regarding the presence of pain or discomfort, activity limitations, and medical consultations related to site-specific musculoskeletal issues [49]. Responses are categorized as “yes” or “no”. The NMQ has shown good validity and is a reliable tool for assessing musculoskeletal symptoms [49, 50].

#### Health - linkage to the National Health-related Lifestyle Survey

We will link participants’ data with the data collected in the National Health-related Lifestyle Survey by the NIJZ. The national survey is a 90-item questionnaire that is available at: https://nijz.si/publikacije/kako-skrbimo-za-zdravje-z-zdravjem-povezan-vedenjski-slog-prebivalcev-slovenije-2020/. For our study, we will obtain the following data on:

- *Demographic information*, including age (in years), sex (male / female), marital status (single / married / engaged in an extramarital affair / widowed / divorced), number of household members (using the question “How many members currently reside in your household?”, with the numerical response provided), number of minor household members (using the question “How many children under the age of 18 currently reside in your household?”, with the numerical response provided), employment status (using the question “What is your employment status?”, with the response options: employed / self-employed / student / farmer/helping family member / retired / unemployed / other), level of education (using the question “What is your highest level of education?”, with the response options: incomplete primary education / primary education / lower or secondary vocational education / secondary vocational, general education / higher vocational education, post-secondary education / higher vocational education (also 1st Bologna cycle) / higher university education (also 2nd Bologna cycle) / specialization, master’s degree, doctorate), and socio-economic position (using the question “To which social class would you say you belong?”, with the response options: lower / middle / upper middle / upper / don’t know).
- *Health status*, including self-rated health (using the question “In general, how would you rate your current health status?”, with the response options: very good / good / fair / bad / very bad), presence of health issues (using the question “Have you had any of the following problems in the last 30 days? followed by a list of health issues: chest pain during physical activity, low back pain, neck/shoulder pain, pain in other joints, persistent coughing fits with mucus, swelling of the legs, allergy, constipation, headache, sleep problems, depressive state (depression, sadness), toothache, difficulty urinating, and anxiety state (extreme worry, fear), with the response options: yes / no), diagnosed health conditions (using the question “Have you been diagnosed with any of the following conditions?” followed by a list of health conditions: high blood pressure over 140/90 mmHg (hypertension), high blood fats (cholesterol and/or triglycerides), diabetes (gestational diabetes not included), previous heart attack or myocardial infarction, chest pain at rest or during physical activity (angina pectoris), heart failure, stroke, spinal diseases and disorders, joint diseases (arthritis or arthrosis), chronic bronchitis, emphysema (COPD), bronchial asthma, gastric or duodenal ulcer, liver cirrhosis, depression, anxiety disorders, other mental illnesses, sleep disorders, thyroid disease, chronic renal failure, and end-stage renal failure, with the response options: yes, diagnosed within the past 12 months / yes, diagnosed over 12 months ago / no), medication usage (using the question “Have you used any of the following medications in the past 7 days?” followed by a list of medications: to lower blood pressure, to lower blood fats (cholesterol and/or triglycerides), to lower blood sugar, for headaches, for other pain, for coughs, sedatives, sleeping pills, nutritional supplements and herbal preparations, contraceptives, antidepressants, homeopathic medicines, and other types of medicines, with the response options: yes / no), stress frequency (using the question “How often have you felt tense, under stress, or great pressure in the past 14 days?”, with the response options: never / very rarely / occasionally / often / every day), stress management (using the question “How often can you find a way to relax when you need to?”, with the response options: never / very rarely / occasionally / often / every day), perceived social support (using the question “How many people can you count on for support with a serious personal problem?”, with the response options: none / 1 or 2 / 3 to 5 / more than 5), body mass index (calculated from self-reported body height (in cm) and body weight (in kg)), and waist circumference (using the question “What is your waist circumference at belly button height?” with the numerical response provided in cm).
- *Health-related behaviours*, including 24-hour movement behaviours (using GIB24-Q, see above), meals frequency (using the question “How many meals (for example, breakfast, mid-morning snack, afternoon snack, lunch, and dinner) do you usually eat per day?”, with the numerical response provided), diet (using the question “How often do you usually eat the following foods?” followed by a list of foods: milk and dairy products (milk, cheese, yogurt, cottage cheese…), fresh fruit, processed fruit (compotes, canned fruit, 100% fruit juice, smoothies…), raw vegetables, processed vegetables (boiled, stewed, preserved…), poultry (chicken or turkey meat), red meat (beef, pork, horse meat), fish and seafood, potatoes, rice, pasta, egg as a separate dish, white, semi-white bread and pastries, whole-grain bread and pastries, cereals (cereals, muesli, porridge), fried foods (french fries, fried meat, fritters…), sandwiches, hot dogs, pizza, burek, kebab, with the response options: never / 1–3 times per month, 1–3 times per week, 4–6 times per week, 1 time per day, 2 times per day or more), smoking (using the question “Do you currently smoke or have you ever smoked tobacco (cigarettes, cigars, cigarillos, or pipe tobacco)?, with the response options: I don’t smoke and have never smoked / I don’t smoke anymore, but I used to / I’m currently a smoker), alcohol (using the question “How often have you drunk beverages containing alcohol (e.g., beer, wine, spirits, liqueur, cider, radler, cocktails) in the last 12 months?”, with the response options: 1 time per month or less / 2–4 times per month / 2–3 times per week / 4 times per week or more).

#### Health - linkage to the national health-related registers

We will link participants’ data with the data from several national registers: healthcare utilisation register, hospitalisation register, drug prescription register, sickness absence register, retirement register, incidence diseases register, and causes of death register. We will submit a formal application to the statistical office of NIJZ that is responsible for collecting and storing personal data (legal basis in the health care databases act (ZZPPZ—Ur. l. RS 65/00) and personal data protection act (ZVOP-1—Ur. l. RS 94/07)). Individual-level data that are collected as part of the national statistics are strictly confidential and may only be used for statistical purposes [51]. When assessing to national health registers, our research team will comply with the General Data Protection Regulation, the Data Protection Act, and the National Statistics Act [51].

### Ethical considerations and data management

Participants will sign informed consent before study enrolment. The first two data collections (in 2024 and 2026) were approved by the National Ethics Committee (Republic of Slovenia National Medical Ethics Committee, ref: 0120 − 62/2024-2711-6). Ethics approval for further data collections will be obtained after successful completion of the first two data collections. Funding for the first baseline data collection (2024) was provided within the project “V3-2305 Implementing the concept of 24-hour movement behaviours as a determinant of health into the Slovenian environment (GIB24)”, funded by Slovenian Research and Innovation Agency and Slovenian Ministry of Health. Funding for the further data collections is jet to be obtained by future projects. Any future protocol modifications will be communicated to study participants (by e-mail or by post letter), and research community (by publishing an updated protocol).

Each participant will be assigned with a study ID that will be used to track participants’ data. Raw data will be securely stored on external hard drive and only principal investigators of the study will have access to the source data. All paper-based documentation (informed consents, questionnaires, diaries) collected during the study will be securely stored in archives of principal investigators’ institutions. Personal data will be collected, processed, and stored in accordance with the principles of the General Data Protection Regulation (GDPR). The study results will be published in peer-reviewed journals and presented at the conferences. Part of the anonymised dataset may be published along research papers. It is anticipated that the longitudinal *GIB study* will join international consortiums focused on epidemiological studies using thigh-worn accelerometers, with the aim to establish pooled data resources on 24-hour movement behaviours and health outcomes.

### Statistical analysis

Statistical analysis will be conducted using R and RStudio with the packages dedicated for compositional data analysis (“compositions”, “Compositional”, “robCompositions”, “zCompositions”), graphics and visualisation (“ggplot2”, “ggtern”, “plotly”, “rgl”, “shiny”, “Ternary”, “tricolore”), and other packages for data handling, inspection, etc. (“boot”, “car”, “data.table”, “dplyr”, “mice”, “performance”, “survey”, “questionr”, “tidyr”, “tidyverse”).

To obtain population estimates, weights will be applied to account for possible non-representativeness of the sample. Participant characteristics will be presented as absolute and relative frequencies (for categorical data), as means and SD (for symmetrically distributed continuous data), as medians and IQRs (for non-symmetrically distributed continuous data), and as compositional means (for compositional data).

A series of regression analyses will be used to explore the associations of 24-hour movement behaviours with health outcomes, socio-demographics, and built environment. Analysis will be adjusted for several confounding variables that will be selected based on their theoretical impact on the relationship between independent and dependent variable. Compositional data analysis will be used to account for the co-dependency of data on 24-hour movement behaviours. Zeroes in the time-use compositions will be handled by using the log-ratio expectation-maximisation algorithm [52]. The missing data will be handled according to the recommendations [53]. Time-use compositions will be expressed as a set of isometric log ratio coordinates (ilrs) before entering the regression models. We will construct two types of time-use compositions from activPAL data: (*i*) posture-based time-use composition, including stepping, standing, sitting, lying while awake, and sleeping, and (*ii*) movement-based time-use composition, including moderate- to vigorous-intensity physical activity, light-intensity physical activity, sedentary behaviour, and sleep. For employed participants, we will also construct domain-specific time-use compositions (i.e., including occupational and non-occupational movement behaviours).

For models with significant findings, we will explore the optimal time-use zones (e.g., time-use zone associated with the best 5% health outcomes [16, 54]). Regression model will be used to estimate predicted health outcome for all possible 24-hour movement behaviour compositions (with 10 min granularity and within the bounds observed in the sample). The zone (range) of 24-hour movement behaviour compositions associated with the top 5% of predicted health outcomes will be described as “the best zone” or the Goldilocks Zone. As it is very likely that optimal time-use compositions will differ for different health outcomes, we will also explore an overlap of best zones for different health outcomes, i.e., the 24-hour movement behaviour compositions for which all health outcomes explored were predicted to be in the best 5% [16, 54]. Special focus will be paid on the relative importance of domain-specific physical activity (i.e., occupational and non-occupational). This will be achieved by constructing a time-use compositions that includes domain-specific components and run the analysis by feeding the models with domain-specific time-use compositions.

## Discussion

### Expected results

*The GIB study* will be the first longitudinal population-based study that will invite a national representative sample of Slovenian adults to provide data on device-measured 24-hour movement behaviours along with self-reported data on health and built environment. The study will allow for the first time to estimate device-measured levels of 24-hour movement behaviours among Slovenian adults, and to explore their socio-demographic and built environment correlates and determinants.

This study will produce new knowledge on the cross-sectional and longitudinal associations between 24-hour movement behaviours and numerous health outcomes including self-reported and register-based data on health, adjusted for a range of confounders and accounted for co-dependency between 24-hour movement behaviours using compositional data analysis. An important strength of the study will be to reveal insight into the relative importance of domain-specific and type-specific physical activity and sedentary behaviour; and will help establish device-measured and self-reported optimal time-use for different health outcomes, informing future 24-hour movement guidelines for adults.

Furthermore, the study will produce new knowledge on the potential mediating effect of physical fitness, diet, and other health-related behaviours on the relationship between 24-hour movement behaviours and health; and to explore the potential moderating effect of the built environment. Finally, this study is anticipated to contribute data to international consortia that collect and analyse pooled thigh-worn accelerometer data resources on 24-hour movement behaviours, posture, and health outcomes.

## Data Availability

No datasets were generated or analysed during the current study.

## Abbreviations

ESS: Epworth Sleepiness Scale
FAS: Fatigue Assessment Scale
GDPR: General Data Protection Regulation
GIB24-Q: Questionnaire on movement behaviours included in Slovenian Health-related Lifestyle Survey
GPAQ: Global Physical Activity Questionnaire
HeBEQ: Healthy Built Environment Questionnaire
IFIS: International Fitness Scale
NIJZ: National Institute of Public Health
NMQ: Nordic Musculoskeletal Questionnaire
NRS: Need for Recovery Scale
PTPS: Perceived Time Poverty Scale
SPIRIT: Standard Protocol Items: Recommendations for Interventional Trials
SWLS: Satisfaction With Life Scale
The GIB study: The prospective study on 24-hour movement behaviours, built environment, and health
